# Trends in motives for attempts to reduce alcohol consumption among risky adult drinkers in England: A representative population survey, 2017–2024^☆^

**DOI:** 10.1016/j.dadr.2025.100340

**Published:** 2025-05-05

**Authors:** Dimitra Kale, Vera Buss, Melissa Oldham, Jamie Brown, Lion Shahab, Sarah Jackson

**Affiliations:** a Department of Behavioural Science and Health, University College London, UK; b SPECTRUM Research Consortium, UK

**Keywords:** Alcohol, Motives to reduce, Risky drinkers, England, drinkers, Trend analysis

## Abstract

**Background:**

Understanding the motives for reducing alcohol consumption, how they differ among various population groups, and how they have evolved over time is crucial for designing effective public health interventions. This study estimated time trends in motives for attempts to reduce alcohol consumption among risky adult drinkers in England between 2017 and 2024 and explored differences by sociodemographics and alcohol consumption levels.

**Methods:**

Data came from a nationally representative survey (Alcohol Toolkit Study), assessing 11,974 risky adult drinkers (mean [Standard Deviation] age= 45.8 [15.7] years, 60.1 % men) who made a past-year reduction attempt between January/2017 and August/2024. Participants reported factors motivating their most recent attempt (not mutually exclusive). We estimated time trends in the proportion of attempts to reduce alcohol consumption motivated by health concerns, cost, social factors (i.e., peer influence/support) and health professional advice, and calculated prevalence ratios (PRs) to compare changes in prevalence across the whole time series.

**Results:**

Over the time series, there was a small increase in the proportion of attempts motivated by health concerns (70.5–76.8 %; PR=1.09, 95 %CI1.01–1.18). Attempts motivated by cost and social factors nearly doubled (10.7–20.2 %; PR=1.89, 95 %CI1.37–2.60; 13.3–25.5 %; PR=1.92, 95 %CI1.46–2.52, respectively). Attempts driven by health professional advice increased (4.4–7.0 %; PR=1.57, 95 %CI0.96–2.57). Women, individuals from less advantaged social grades and with AUDIT-C 5–7 exhibited more pronounced changes in the proportion of attempts motivated by health concerns. Cost was a more consistent driver of attempts for those aged≥ 65.

**Conclusions:**

Health concerns remain the most common motive for attempts to reduce alcohol consumption, but the proportion of attempts motivated by cost and social factors nearly doubled over the study period. These findings suggest the need for public health interventions that consider both economic and social influences alongside health concerns to better support alcohol reduction.

## Highlights

• Health concerns remain the most common motive for attempts to reduce alcohol consumption.
• Over the study period, the proportion of attempts motivated by cost and social factors nearly doubled.
• Attempts to reduce alcohol consumption linked to health professional advice showed an uncertain increase and was the least common motive.

## 1 Introduction

There has been an increase in the proportion of people who drink alcohol at risky levels (operationalised as an Alcohol Use Disorders Identification Test Consumption (AUDIT-C) score ≥ 5 (Bush et al., 1998) motivated to reduce their consumption in England since the Covid-19 pandemic (Buss et al., 2024, Jackson et al., 2021). Understanding the motives behind efforts to reduce alcohol consumption, how they differ among various population groups, and how they have evolved over time is crucial for developing effective public health interventions.

Traditionally, motives to reduce alcohol intake have been driven by health concerns, such as current and future health problems and improving fitness (Epler et al., 2009, Pennay et al., 2019, Beard et al., 2017, Britton and Bell, 2015). Social factors such as peer influence and family support, along with financial concerns and advice from health professionals, also play a role (Epler et al., 2009, Pennay et al., 2019, Beard et al., 2017, Britton and Bell, 2015). A general population study in England from 2014 to 2016 found that individuals with AUDIT-C scores greater than seven were primarily motivated by health concerns, advice from healthcare professionals, and financial reasons to reduce alcohol intake, with higher levels of alcohol consumption linked to these motives (Beard et al., 2017). Motives varied widely, with a quarter not selecting any specific reasons for their behaviour change (Beard et al., 2017).

Recent events, including public health campaigns, increasing awareness of alcohol-related harms, the Covid-19 pandemic and a cost-of-living crisis may have influenced motives for reducing alcohol consumption. Research on the cost-of-living crisis showed that cost-motivated alcohol reduction attempts increased between 2021 and 2022 in Great Britain (Jackson et al., 2023), particularly among individuals from less advantaged social grades. However, this study did not find any significant overall increase in the prevalence of alcohol reduction attempts, suggesting that while cost is becoming a more common motivator, it has not led to a substantial rise in the total number of people trying to reduce their consumption (Jackson et al., 2023). On the other hand, opposing forces such as stress and declining mental health, exacerbated by the pandemic and cost-of-living crisis, may have weakened health-related motives for reducing alcohol consumption. According to the Extended Parallel Process Model (Popova, 2012), individuals may recognise the risks of drinking but struggle with low self-efficacy (i.e. doubt their ability to reduce alcohol consumption), leading them to ignore health messages rather than take action to reduce alcohol consumption.

Motives to reduce alcohol consumption also vary across different sociodemographic groups. For example, older individuals often cite health reasons, while younger adults focus more on lifestyle or social influences (Pennay et al., 2019, Britton and Bell, 2015). Women are generally more likely than men to report health-related motivations (Bernards et al., 2009, Britton and Bell, 2015), and financial concerns are particularly important among socioeconomically less advantaged groups (Jackson et al., 2023, Britton and Bell, 2015). These differences may have been exacerbated by the Covid-19 pandemic. Older adults, at higher risk of severe illness from the virus, may have developed stronger health-related motives, a pattern observed in motives for trying to stop smoking (Sarah E Jackson et al., 2024). Conversely, younger adults, who faced greater social and financial disruptions during the pandemic (Clark et al., 2021, Etheridge and Spantig, 2022, Fancourt et al., 2021), might be more influenced by economic factors. Additionally, women, especially those who carried a disproportionate burden of caregiving during the pandemic, may have developed unique social and health-related motives (Etheridge and Spantig, 2022, Ayittey et al., 2020).

Motives for reducing alcohol consumption are not static and may have evolved in response to recent societal challenges. Understanding current motives for reducing alcohol consumption is crucial for designing targeted interventions that align with individual goals, increasing the effectiveness of the support provided. This study aimed to address the following research questions:

1. How have the proportions of attempts to reduce alcohol consumption that are motivated by (a) health concerns (including Covid-19), (b) cost, (c) social factors and (d) health professional advice changed between 2017 and 2024?
2. To what extent have changes in motives for attempts to reduce alcohol consumption differed by age, gender, social grade, and level of alcohol consumption?

## 2 Methods

### 2.1 Pre-registration

The study protocol and analysis plan were pre-registered on Open Science Framework (https://osf.io/w5kjp).

### 2.2 Design

Data were drawn from the ongoing Alcohol Toolkit Study (ATS), a monthly cross-sectional survey of a representative sample of adults in England (Fidler et al., 2011, Beard et al., 2015). The study uses a hybrid of random probability and simple quota sampling to select a new sample of approximately 1700 adults each month. Full details of the sampling procedure are provided elsewhere (Kock et al., 2021, Beard et al., 2015).

Data were initially collected through face-to-face computer-assisted interviews. However, social distancing restrictions under the Covid-19 pandemic meant no data were collected in March 2020 and data from April 2020 onwards have been collected via telephone. The telephone-based data collection used similar sampling and weighting approaches as the face-to-face interviews. Comparisons of the two data collection modalities indicate broad comparability (Kock et al., 2021).

Interviews were held with one member of each household. Informed consent was obtained prior to each interview. Ethical approval was provided by UCL’s Research Ethics Committee (0498/001). Participants provide informed consent to take part in the study, and all methods are carried out in accordance with relevant regulations. The data are not collected by University College London (UCL) and are anonymised when received by UCL.

### 2.3 Study sample and recruitment

Data included in the present study were collected from respondents surveyed between January 2017 (three years before the start of the Covid-19 pandemic) through to August 2024 (the most recent data at the time of analysis). However, since April 2022, data on the motives for the most recent attempt to reduce alcohol consumption have not been collected in every wave due to availability of funding. Data were not collected for the following months: May, July, September, November, and December/2022, July, September, and November/2023, and May and July/2024. These months were excluded from the analysis.

We used data from participants (≥18 years old), who reported risky drinking operationalised as an AUDIT-C score ≥ 5 (1) (‘risky drinkers’) and having made at least one attempt to reduce their alcohol consumption in the past year.

### 2.4 Measures

Past-year attempts to cut down on drinking alcohol were assessed with the question: ’How many attempts to restrict your alcohol consumption have you made in the last 12 months (e.g., by drinking less, choosing lower strength alcohol or using smaller glasses)? Please include all attempts you have made in the last 12 months, whether or not they were successful, and any attempt that you are currently making.’ Only those replied ≥ 1 attempts to this question were included in the analytic sample.

### 2.5 Outcomes

Motives for the most recent attempt to reduce alcohol consumption were assessed with the question: ‘Which of the following if any, do you think contributed to you making the most recent attempt to restrict your alcohol consumption?’ Participants could select multiple motives from a list of options: (a). Advice from a doctor/health worker, (b). Government TV/radio/press advert, (c). A decision that drinking was too expensive, (d). I knew someone else who was cutting down, (e). Health problems I had at the time, (f). A concern about future health problems, (g). Something said by family/friends/children, (h). A significant birthday or event, (i). Improve my fitness, (j). Help with weight loss, (k). Detox, (l). Had a baby/pregnancy, (m). Family problems, (n). The coronavirus outbreak (from April 2020 onwards), (o). Other (please specify), Not stated, Don’t know/can’t remember.

We reported descriptive data on each motive, aggregated across the study period. Time trends were explored for the following motives, which have been identified in previous studies as the most popular reasons for trying to cut down alcohol consumption (Beard et al., 2017; Britton and Bell, 2015; Epler et al., 2009; Jackson et al., 2024; Pennay et al., 2019):

i) health concerns (e, f, i, j, k, l, n), ii) cost (c), iii) social factors (d, g, h, m), and iv) health professional advice (a).

#### 2.5.1 Covariates

Sociodemographic characteristics included age (18–24, 25–34, 35–44, 45–54, 55–64, ≥65 years), gender (man, woman, identified in another way; owing to the small proportion of participants who identified as ‘in another way’, they were excluded from the regression analyses), and occupational social grade (AB=higher and intermediate managerial, administrative and professional, C1 =supervisory, clerical and junior managerial, administrative and professional, C2 =skilled manual workers, D=semiskilled and unskilled manual workers, E = state pensioners, casual and lowest-grade workers, unemployed with state benefits only) (Collis, 2009).

Level of alcohol consumption based on AUDIT-C score (Bush et al., 1998) was categorised as AUDIT-C 5–7 and AUDIT-C> 7.

### 2.6 Statistical analysis

Data were analysed using RStudio v.4.2.2. Missing cases were excluded on a per-analysis basis. Surveys without data collection on motives were excluded from the analysis. However, as we modelled time used restricted cubic splines, we were able to extract estimates for each month from the model, including those in which no data collection took place.

Descriptive statistics were calculated to characterise the sample. For all analyses data were weighted to match the population in England on the dimensions of age, social grade, region, housing tenure, ethnicity, and working status within sex. This profile is determined each month based on data from the 2011 and 2021 UK Census, the Office for National Statistics mid-year estimates, and the annual National Readership Survey (Fidler et al., 2011).

We report descriptive data on the proportion of participants, aggregated across all survey waves, who endorsed each motive for trying to reduce alcohol consumption and the proportion reporting key motives (health concerns, cost, social factors and health professional advice) by age, gender, social grade and level of alcohol consumption.

Logistic regression models were used to test the association of key motives with survey month. Survey month was modelled using restricted cubic splines with five knots, to allow relationships with time to be flexible and non-linear, while avoiding categorisation (Harrell, 2015).

To explore moderation by age, gender, social grade and levels of alcohol consumption, we repeated the models including the interaction between the moderator of interest and survey month, thus allowing for time trends to differ across sub-groups. Each of the interactions was tested in a separate model. We used predicted estimates from our models to plot the prevalence of each outcome over the study period (overall and by moderating variables), alongside raw (weighted) data, and report prevalence ratios (PR) for the change in prevalence across the whole time-series (August/2024 versus January/2017) alongside 95 % Confidence Intervals (CI) calculated using bootstrapping (n = 1000).

In a sensitivity analysis, we analysed current health problems and future health concerns separately to explore any differences. Additionally, we conducted an unregistered analysis to assess ‘improve my fitness’ and ‘help with weight loss’ individually, as a high proportion of participants selected these two factors as motives for reducing alcohol consumption. In an unregistered analysis, we also calculated PR for ‘other’ and ‘not stated’ motives, as we observed an overall increase in reported motives for attempts to reduce alcohol consumption. Finally, we repeated the analysis having the coronavirus outbreak as a separate category and including only waves in which this response option was included (from April 2020 onwards).

## 3 Results

In total, the dataset included 11,974 (unweighted; 11,919 weighted) participants (weighted mean [Standard Deviation] age= 45.8 [15.7] years, 60.1 % men), who drank at risky level and reported having made at least one attempt to reduce alcohol consumption in the past year (sociodemographic characteristics and level of alcohol consumption of the sample (%, N) are shown in Table 2).

### 3.1 Overall estimates of motives for attempts to reduce alcohol consumption

The most reported motive for participants’ most recent attempt to reduce alcohol consumption was health concerns (72.3 %); specifically improve fitness (37.9 %), weight loss (36.1 %), concerns about future health (31.5 %) and current health (10.7 %). Social factors were the second most frequent motive (17.3 %), followed by cost (12.8 %) and health professional advice (6.3 %, see Table 1).

**Table 1:** Proportion reporting each factor as a motive driving the most recent attempt to reduce alcohol consumption.

Motive*	**% (95 %CI)**
**Health concerns**	
Health problems I had at the time	10.7 (10.2, 11.3)
A concern about future health problems	31.5 (30.7, 32.3)
Improve my fitness	37.9 (37.0, 38.8)
Help with weight loss	36.1 (35.2, 37.0)
Detox	15.1 (14.5, 15.8)
Had a baby / pregnancy	0.5 (0.4, 0.7)
The coronavirus outbreak^a^	7.7 (7.1, 8.3)
Any health concern	72.3 (71.5, 73.2)
**Cost**	
A decision that drinking was too expensive	12.8 (12.1, 13.4)
**Social factors**	
I knew someone else who was cutting down	8.1 (7.6, 8.6)
Something said by family/friends/children	8.8 (8.3, 9.3)
A significant birthday or event	3.4 (3.0, 3.7)
Family problems	0.1 (0.0, 0.1)
Any social factor	17.3 (16.7, 18.0)
**Health professional advice**	
Advice from a doctor/health worker	6.3 (5.8, 6.7)
**Other reasons**	
Government TV/radio/press advert	2.4 (2.2, 2.7)
Just decided to reduce alcohol consumption	1.1 (0.9, 1.3)
**Don’t know/can’t remember**	1.2 (0.9, 1.4)
**Not stated**	8.5 (8.0, 9.0)
**Other**	6.5 (5.9, 7.0)

Data are aggregated across the study period (January/2017 – August/2024; unweighted n = 11974) and weighted (n = 11919) to match the population in England. *Motives are not mutually exclusive, as participants were allowed to report multiple motives. ^a^ Among participants surveyed from April/2020 onwards (unweighted n = 7958; weighted=7669). CI, confidence interval.

Table 2 presents the proportion of key motives for attempts to reduce alcohol consumption reported across participant subgroups. Health concerns were consistently the most common motive reported by three-quarters of participants (range 60.1–76.4 %). Health concerns were more frequently reported by individuals aged 45–54 years, women (versus men) and those in more (versus less) advantaged social grades. Individuals with AUDIT-C> 7 were also more likely to report health concerns as a motive for their most recent attempt to reduce alcohol consumption than those with AUDIT-C 5–7. Social factors were more frequently reported by younger individuals, those from mid-range social grades (C2, D) and those with AUDIT-C> 7 (versus AUDIT-C 5–7). Cost was more frequently reported as a motive by younger participants, those from less (versus more) advantaged social grades and those with AUDIT-C> 7 (versus AUDIT-C 5–7). Health professional advice was more frequently reported by older individuals, men (versus women), those from less (versus more) advantaged social grades and those with AUDIT-C> 7 (versus AUDIT-C 5–7).

**Table 2:** Sample characteristics and proportion reporting key motives driving the most recent attempt to reduce alcohol consumption by participant characteristics.

		Motive* , % (95 % CI)			
	% (N)	Health concerns	Cost	Social factors	Health professional advice
**Age**					
18–24	11.3 (1348)	67.0 (64.5, 69.5)	27.0 (24.6, 29.4)	22.4 (20.1, 24.6)	4.6 (3.5, 5.7)
25–34	17.0 (2026)	72.2 (70.3, 74.2)	17.6 (16.0, 19.3)	19.8 (18.1, 21.6)	4.6 (3.7, 5.5)
35–44	18.6 (2216)	75.7 (73.9, 77.5)	10.8 (9.5, 12.1)	17.7 (16.1, 19.4)	5.8 (4.8, 6.8)
45–54	22.5 (2685)	76.4 (74.7, 78.0)	9.6 (8.5, 10.8)	18.2 (16.8, 19.7)	6.4 (5.5, 7.3)
55–64	17.0 (2027)	72.9 (71.0, 74.8)	8.5 (7.3, 9.7)	14.4 (12.9, 16.0)	7.8 (6.6, 8.9)
≥ 65	13.6 (1617)	65.0 (62.7, 67.3)	8.1 (6.7, 9.4)	11.3 (9.7, 12.9)	8.5 (7.1, 9.8)
**Gender**					
Man	60.1 (7159)	71.3 (70.2, 72.3)	12.7 (11.9, 13.5)	17.0 (16.1, 17.9)	7.4 (6.8, 8.0)
Woman	39.4 (4692)	74.2 (72.9, 75.4)	12.7 (11.7, 13.7)	17.6 (16.5, 18.7)	4.6 (4.0, 5.2)
Identified in another way	0.5 (55)	60.1 (46.8, 73.5)	27.7 (15.4, 40.0)	33.2 (20.3, 46.2)	3.7 (−1.5, 8.9)
**Social grade**					
AB (most advantaged)	37.1 (4422)	76.3 (75.0, 77.5)	8.5 (7.7, 9.3)	16.0 (14.9, 17.1)	5.3 (4.7, 0.6)
C1	31.7 (3731)	72.5 (71.1, 73.9)	12.9 (11.8, 14.0)	17.3 (16.1, 18.6)	5.4 (4.7, 6.2)
C2	17.3 (2063)	69.1 (67.1, 71.1)	16.3 (14.7, 17.9)	18.9 (17.2, 20.6)	7.1 (6.0, 8.3)
D	9.7 (1153)	67.1 (64.4, 69.8)	18.9 (16.6, 21.1)	19.3 (17.0, 21.6)	7.0 (5.6, 8.5)
E (least advantaged)	4.6 (550)	63.0 (59.0, 67.1)	20.2 (16.8, 23.6)	17.5 (14.3, 20.8)	14.8 (11.8, 17.8)
**Level of alcohol consumption**					
AUDIT-C 5–7	59.9 (7142)	71.01 (70.0, 72.1)	11.8 (11.1, 12.6)	15.7 (14.8, 16.5)	5.4 (4.9, 5.9)
AUDIT-C> 7	40.1 (4777)	74.3 (73.1, 75.6)	14.2 (13.2, 15.2)	19.8 (18.7, 20.9)	7.6 (6.8, 8.4)

Data are aggregated across the study period (January/2017 – August/2024; unweighted n = 11974) and weighted (n = 11919) to match the population in England. *Motives are not mutually exclusive, as participants were allowed to report multiple motives. There were 13 missing cases for gender, valid percentages are presented. CI confidence intervals.

### 3.2 Time trends in motives for attempts to reduce alcohol consumption

**Fig. 1:**
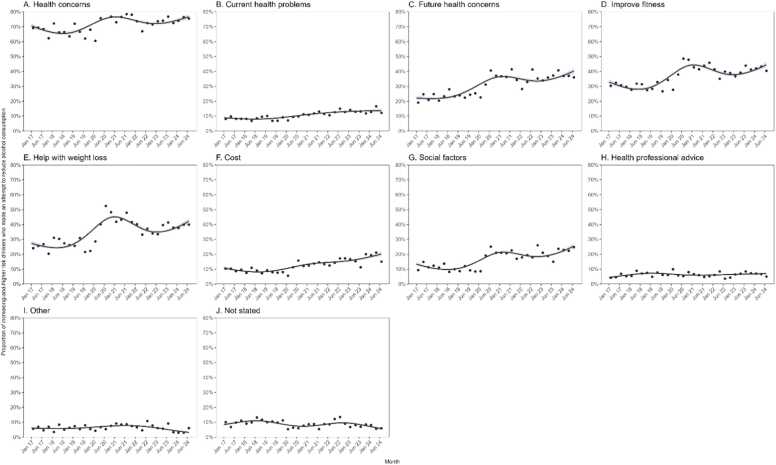
Time tends in motives for attempts to reduce alcohol consumption, January/2017 to August/2024. Panels show the proportion of risky drinkers in England reporting that their most recent attempt to reduce alcohol consumption was motivated by (A) health concerns (current health problems, future health problems, improve fitness, weight loss, detox, the coronavirus outbreak or pregnancy), (B) current health problems, (C) future health concerns, (D) improve fitness, (E) help with weight loss, (F) cost, (G) social factors, (H) health professional advice, (I) Other, (J) Not stated. Lines represent modelled weighted prevalence by survey month, modelled non-linearly using restricted cubic splines (five knots). Shaded bands represent standard errors. Points represent observed quarterly weighed prevalence.

Fig. 1 shows modelled monthly trends in motives for attempts to reduce alcohol consumption and Table 3 compares modelled estimates from the first (January/2017) and last (August/2024) month in the time series. Up until the start of 2020, two in three attempts to reduce alcohol consumption were motivated by health concerns (mean modelled monthly proportion: January/2017 to December/2019 =67.6 %). Specifically, one in three were motivated by improve fitness (30.5 %), one in four by weight loss (27.1 %) and future health (23.8 %), and one in eight by current health (8.4 %). A smaller proportion were motivated by social factors (11.3), cost (9.0 %) and health professional advice (6.4 %).

**Table 3:** Modelled weighted estimates of the proportion of attempts to reduce alcohol consumption motivated by health concerns, cost, social factors and health professional advice in January/2017 and August/2024.

	Prevalence, %*		
Motive	January 2017	August 2024	Prevalence ratio (95 % CI)^ⴕ^
Health concerns	70.5	76.8	1.09 (1.01–1.18)
Current Health	8.8	13.8	1.56 (1.10–2.23)
Future Health	22.1	40.3	1.82 (1.51–2.19)
Improve my fitness	32.9	44.4	1.35 (1.17–1.55)
Help with weight loss	27.3	42.1	1.54 (1.33–1.79)
Cost	10.7	20.2	1.89 (1.37–2.60)
Social factors	13.3	25.5	1.92 (1.46–2.52)
Health professional advice	4.4	7.0	1.57 (0.96–2.57)
Other	6.0	3.2	0.53 (0.32–0.86)
Not stated	8.4	5.7	0.68 (0.46–1.01)

Data from January/2017 and August/2024 are weighted estimates of prevalence in these months (the first and last in the study period) from log-binomial regression with survey month modelled non-linearly using restricted cubic splines with five knots. ^ⴕ^ Prevalence ratio for the change in prevalence across the whole time series (January/2017 versus August/2024) with 95 % confidence intervals calculated using bootstrapping. CI, confidence interval.

When examining time trends with Covid-19 as a separate category and including only waves in which this response option was included (from April/2020 onwards), the proportion reporting Covid-19 as a motive for the most recent attempt to reduce alcohol consumption remained stable until April/2021 and then declined to 3.0 % in December/2022 and has remained low since then (supplementary figure 1).

The proportion of attempts to reduce alcohol consumption motivated by health concerns increased over the study period from 70.5 % in January/2017–76.8 % in August/2024 (PR=1.09, 95 % CI 1.01–1.18; Table 3). This increase, however, was not linear. The proportion reporting health concerns as a motive for their most recent attempt to reduce alcohol consumption increased through 2020 and 2021 (peaking at 76.6 % in February/2021), then declined slightly in 2022 until mid-2023, before increasing again, returning to the 2021 peak (Fig. 1). This pattern was largely driven by future health concerns, improve fitness and weight loss (Figs. 1B, 1D, 1E, respectively). Time trends differed by gender (interaction p = 0.045), social grade (interaction p = 0.028) and levels of alcohol consumption (interaction p = 0.039). Women, individuals from less advantaged social grades (C2, D, E) and those with AUDIT-C 5–7 showed more pronounced changes compared to men, those from more advantaged social grades and those with AUDIT-C> 7 (supplementary figures S2B, C, D).

The proportion of attempts motivated by cost increased significantly from 10.7 % in January/2017–20.2 % in August/2024 (PR=1.89, 95 %CI 1.37–2.60; Table 3). It remained relatively stable until 2019, after which it steadily increased beginning in 2020 (Fig. 1F). Time trends differed significantly by age (interaction p = 0.041), with cost-motivated attempts fluctuating more among older (≥65) individuals, but showed a fairly steady increase among those < 65 (supplementary figure 3A).

There was a significant increase in the proportion of attempts to reduce alcohol consumption motivated by social factors, rising from 13.3 % in January/2017–25.5 % in August/2024 (PR=1.92, 95 %CI 1.46–2.52; Table 3). This rise was non-linear, with the proportion remaining stable until the end of 2019, then increasing in 2020 (peaking at 21.4 % in March/2021). Afterwards, it declined slightly in 2021 and 2022, before increasing again from mid-2023 (Fig. 1G). No significant interaction was observed with age, gender, social grade and alcohol consumption levels (supplementary figure 4).

There was an uncertain increase in the proportion of attempts to reduce alcohol consumption that were motivated by health professional advice, rising from 4.4 % in January/2017–7.0 % in August/2024 (PR=1.57, 95 %CI 0.96–2.57). Again, the trend was non-linear, with a peak at 7.3 % in February/2019, followed by a decline to 5.9 % by the end of 2020 and then a steady increase thereafter. No significant interaction was observed with age, gender, social grade and alcohol consumption levels (supplementary figure 5).

The proportion of attempts reporting ‘other motives’ decreased from 6.0 % in January/ 2017–3.2 % in August/2024 (PR=0.53, 95 %CI 0.32–0.86; Table 3), while the proportion reporting ‘no stated’ motives decreased from 8.4 % to 5.7 % (PR=0.68, 95 %CI 0.46–1.01; Table 3).

## 4 Discussion

Between 2017 and 2024 in England, adults drinking at risky levels reported various reasons for attempting to reduce their alcohol consumption. Consistent with previous research (Epler et al., 2009, Pennay et al., 2019, Beard et al., 2017, Britton and Bell, 2015), health concerns were the most frequently cited motivation. Other commonly reported factors included cost and social factors, such as pressure from family and friends, while health professional advice was less commonly reported. Over the years, the relative prevalence of these motives changed. Before 2020, approximately two-thirds of attempts were motivated by health concerns, with specific reasons including improve fitness, future health concerns, weight loss, and current health. Social factors were a motive in about one in nine attempts, cost in one in eleven and health professional advice in one in 15. In 2020, a notable increase occurred in the proportion of attempts to reduce alcohol consumption motivated by health concerns, social factors, cost, and health professional advice. For health concerns, this increase peaked in 2021, followed by a slight decline in 2022 and then rose again, reaching peak levels in 2024. Conversely, the proportion of attempts to reduce alcohol consumption motivated by social factors and cost continued to increase (reaching 25.2 % and 20.2 %, respectively in August/2024) and almost doubled during the study period. The proportion of attempts to reduce alcohol consumption motivated by health professional advice showed an uncertain increase over time but remained low in absolute terms throughout the study period. The proportion of attempts reporting ‘other’ or ‘not stated’ motives decreased over the study period.

Many of these changes are likely attributable to the Covid-19 pandemic, which began affecting England in March/2020. The timing of the pandemic’s onset coincided with increases in the proportion of participants reporting health concerns, social factors and cost as motives for attempting to reduce alcohol consumption. Health concerns, already a prevalent motive, became even more prominent during the pandemic’s first year. The increase in health concerns as a motive for attempts to reduce alcohol consumption was particularly pronounced among women, who are generally more concerned about their weight than men (Tsai et al., 2016) and thus may have been more likely to worry about weight gain during lockdown periods when outdoor activities were limited. This increase was also more pronounced among those from less advantaged social grades, who experienced higher rates of Covid-19 cases (Statistics, 2021) and among individuals with AUDIT-C levels less than seven. Once lockdown restrictions were lifted, and the immediate threat of the virus was mitigated through vaccination programmes, a slight decline in health-related motives was observed, followed by a resurgence in mid-2023, returning to levels seen during the pandemic’s peak.

Our findings also indicate a rise in attempts motivated by social factors during the pandemic. As drinking increasingly took place at home due to closure of bars and other on-premises drinking establishments, alcohol consumption may have become more visible to partners and family members. This increased visibility could have led to greater social pressure or concerns being raised by loved ones, prompting some individuals to attempt to reduce their alcohol consumption. Additionally, caring for children at home may have motivated some to avoid drinking in their presence. Furthermore, health risks associated with Covid-19 may have encouraged individuals to support loved ones in reducing alcohol consumption, while some people may have been influenced to reduce alcohol consumption by those around them making similar attempts.

The proportion of attempts to reduce alcohol consumption motivated by cost remained relatively stable until 2019, followed by a steady increase. This rise may have initially been driven by the economic impact of Covid-19, which caused job losses and income reduction for many (Nicola et al., 2020). The rising cost of essentials becoming a significant factor from late 2021 onward. Similarly, research on the cost-of-living crisis found an increase in attempts to reduce alcohol consumption motivated by cost between December/2021 and December/2022 among risky drinkers from less advantaged social grades (Jackson et al., 2023). Our findings extend this observation, showing that the trend continued through August/2024.

The pandemic significantly impacted healthcare delivery (BMA, 2023), leading to a shift toward remote consultations and prioritisation of acute care over preventive and health promotion services. Additionally, many patients often delayed seeking healthcare, contributing to a decline in attempts to reduce alcohol consumption motivated by health professional advice in 2020, a trend that continued through 2024. This and previous studies consistently show that small proportions of risky drinkers receive an offer of support (Kock et al., 2024, Buss et al., 2023). The main barriers identified by healthcare practitioners to delivering brief alcohol interventions include lack of time, insufficient training and beliefs about the inability to effectively deliver screening and advice (Johnson et al., 2010, Rosário et al., 2021). These findings suggest missed opportunities to promote alcohol reduction in healthcare settings in part due to time constraints and healthcare professionals' lack of confidence (Barry et al., 2004). This is particularly concerning as brief interventions can be effective (Beyer et al., 2018).

### 4.1 Implications

These findings have several important implications for public health policy and intervention strategies. Given the importance of fitness and weight loss as motives in attempts to reduce alcohol consumption, policies placing calorie information on alcohol products could encourage more individuals to reconsider their drinking habits. A recent study found that nearly half of alcohol consumers would change their drinking habits if calorie labels were introduced (Steptoe et al., 2024). While calorie labels alone may not drastically change drinking behaviours, it could be a valuable component of a broader public health strategy alongside regulations on advertising, availability, and pricing. Additionally, a recent World Health Organisation-Europe report recommends that alcohol labels should warn about cancer risk, empowering consumers to make informed choices that could help reduce alcohol-related harms (Europe, 2025).

The growing importance of cost as a motive for attempts to reduce alcohol consumption highlights the potential effectiveness of campaigns emphasising the financial savings associated with reduced alcohol consumption. Additionally, policies ensuring that alcohol-free or low alcohol drinks are sold at a lower price than standard strength alcoholic drinks may further support individuals motivated by cost to reduce their alcohol consumption (Oldham et al., 2024).

Findings also indicate that women, individuals from less advantaged social grades and those with moderate alcohol consumption exhibited more pronounced changes in their attempts to reduce alcohol consumption motivated by health concerns. These findings suggest the need for tailored public health interventions. For example, campaigns targeting women could focus on fitness and weight management, while campaigns for individuals with moderate alcohol consumption could raise awareness of potential long-term health risks and encourage early intervention.

### 4.2 Strengths and limitations

This study had a large, representative, monthly sample, enabling detailed analysis of trends over time. However, several limitations should be acknowledged. First, the study relied on self-reported data based on participants’ recall of the previous year. This may have led participants, particularly those whose attempts began further in the past, to recall only the most meaningful motive that contributed to their attempt. Second, participants, who successfully reduced their drinking, having now an AUDIT-C scores below five, would not be asked about their motives to reduce alcohol consumption. Third, the mode of data collection shifted from face-to-face to telephone interviews in April 2020 and even though comparisons of the two data collection modalities indicate broad comparability (Kock et al., 2021), lower levels of alcohol consumption were observed in face-to-face interviews compared to telephone interviews (Kock, et al., 2024). This could partially explain the observed increases in motives during this period. However, since most increases were temporary and subsequent changes occurred despite no further methodological adjustments, these changes are more likely attributable to other factors rather than the change in data collection methods. Additionally, the steady increase in attempts motivated by current health problems, along with the temporary rise in motives related to future health concerns and weight loss during the early days of the pandemic, may also suggest that these trends reflect genuine changes, though artefactual changes due to shift in data collection method cannot be excluded. Fourth, the findings may not be generalisable to all drinkers as only risky drinkers are included in this study and household surveys often underrepresent dependent drinkers (Rehm et al., 2021). Moreover, results might not apply to other countries with different cultural attitudes towards drinking, alcohol regulations or support systems. Lastly, while the survey captured a broad range of motives and provided descriptive data on all of them, trend analyses were conducted to only the four most prevalent motives based on prior literature (Beard et al., 2017). This may have overlooked trends in less commonly reported motives.

### 4.3 Conclusions

In conclusion, health concerns remain the most common motive for attempting to reduce alcohol consumption among individuals who drink at risky levels. Over the study period, the proportion of attempts motivated by health concerns generally increased, with those motivated by cost and social factors nearly doubling. Attempts to reduce alcohol consumption linked to health professional advice showed an uncertain increase and was the least common motive. Policies that incorporate calorie, weight, and cost-related information in public health campaigns could effectively help individuals make informed decisions about their alcohol consumption.

## CRediT authorship contribution statement

**Kale Dimitra:** Writing – review & editing, Writing – original draft, Methodology, Formal analysis, Data curation, Conceptualization. **Oldham Melissa:** Writing – review & editing, Conceptualization. **Buss Vera Helen:** Writing – review & editing, Conceptualization. **Brown Jamie:** Writing – review & editing, Funding acquisition, Conceptualization. **Jackson Sarah:** Writing – review & editing, Supervision, Conceptualization. **Shahab Lion:** Writing – review & editing, Funding acquisition, Conceptualization.

## Ethics approval

Ethical approval for the STS was granted originally by the UCL Ethics Committee (ID 0498/001). The data are not collected by UCL and are anonymized when received by UCL.

## Authors disclosure

DK, VB, SJ, MO declare no conflicts of interest. JB has received unrestricted research grants from Pfizer related to smoking cessation.LS has received honoraria for talks, an unrestricted research grant and travel expenses to attend meetings and workshops from Pfizer and has acted as paid reviewer for grant awarding bodies and as a paid consultant for health care companies.

## Funding

This work was supported by 10.13039/501100000289CRUK (PRCRPG-Nov21\100002). For the purpose of Open Access, the author has applied a CC BY public copyright licence to any Author Accepted Manuscript version arising from this submission

## Declaration of Competing Interest

JB has received unrestricted research funding from Pfizer and J&J, who manufacture smoking cessation medications. LS has received honoraria for talks, unrestricted research grants and travel expenses to attend meetings and workshops from manufactures of smoking cessation medications (Pfizer; J&J) and has acted as paid reviewer for grant awarding bodies and as a paid consultant for health care companies. All authors declare no financial links with alcohol companies or their representatives.
