# Supplementary material for: Trends in motives for attempts to reduce alcohol consumption among risky adult drinkers in England: A representative population survey, 2017–2024
Source: Drug Alcohol Depend Rep. 2025 May 5;15:100340. doi: 10.1016/j.dadr.2025.100340 (PMC12136797; doi:10.1016/j.dadr.2025.100340)
Supplement: Supplementary file 1 — Supplementary material [file mmc1.docx]

Supplementary Material


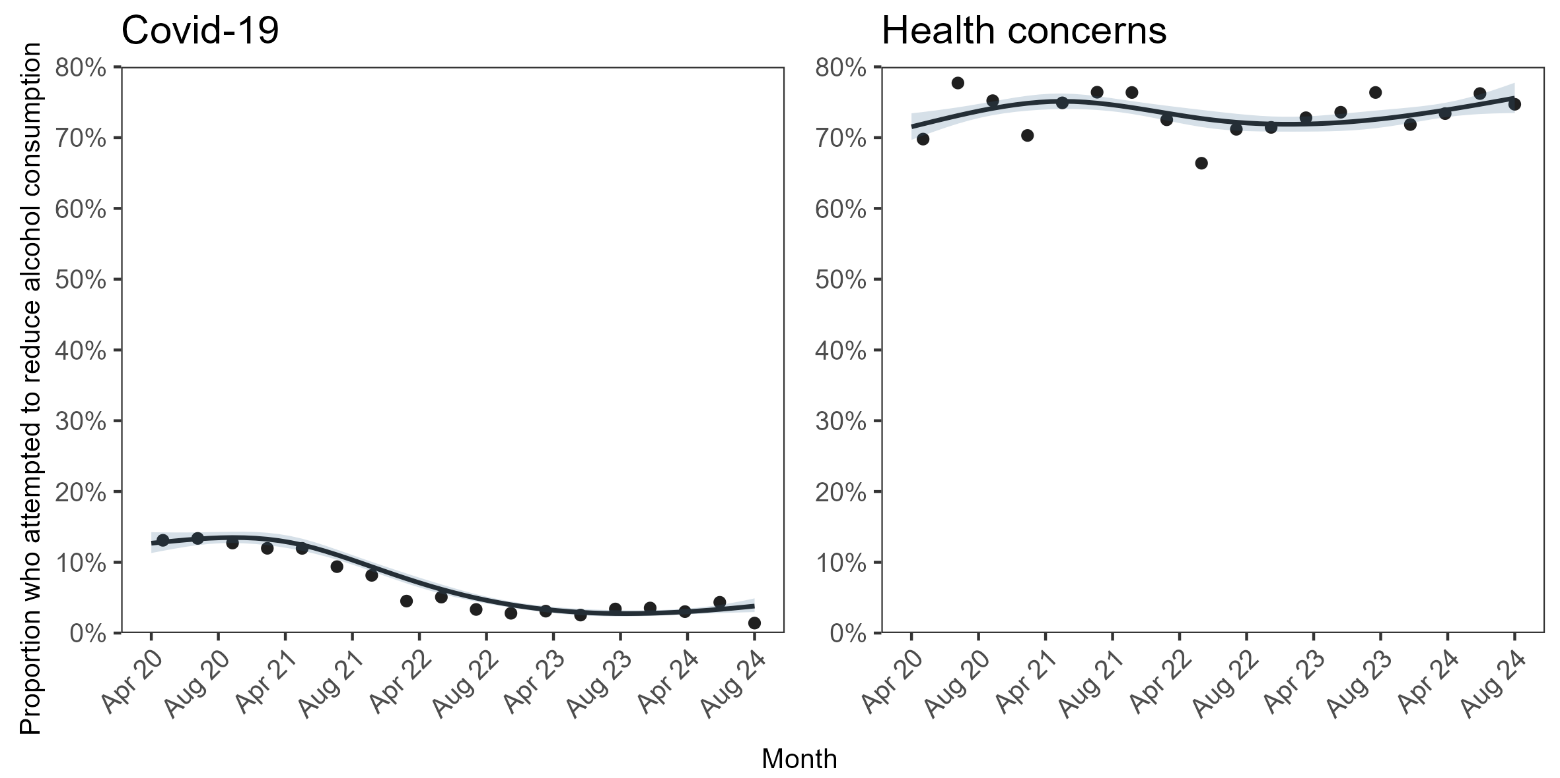


S1 Figure. Time tends in motives for attempts to reduce alcohol consumption, April/2020 to August/2024. Panels show the proportion of increasing-and-higher risk drinkers in England reporting that their most recent attempt to reduce alcohol consumption was motivated by Covid-19 and Health concerns (excluding Covid-19). Lines represent modelled weighted prevalence by survey month, modelled non-linearly using restricted cubic splines (five knots). Shaded bands represent standard errors. Points represent observed quarterly weighed prevalence.


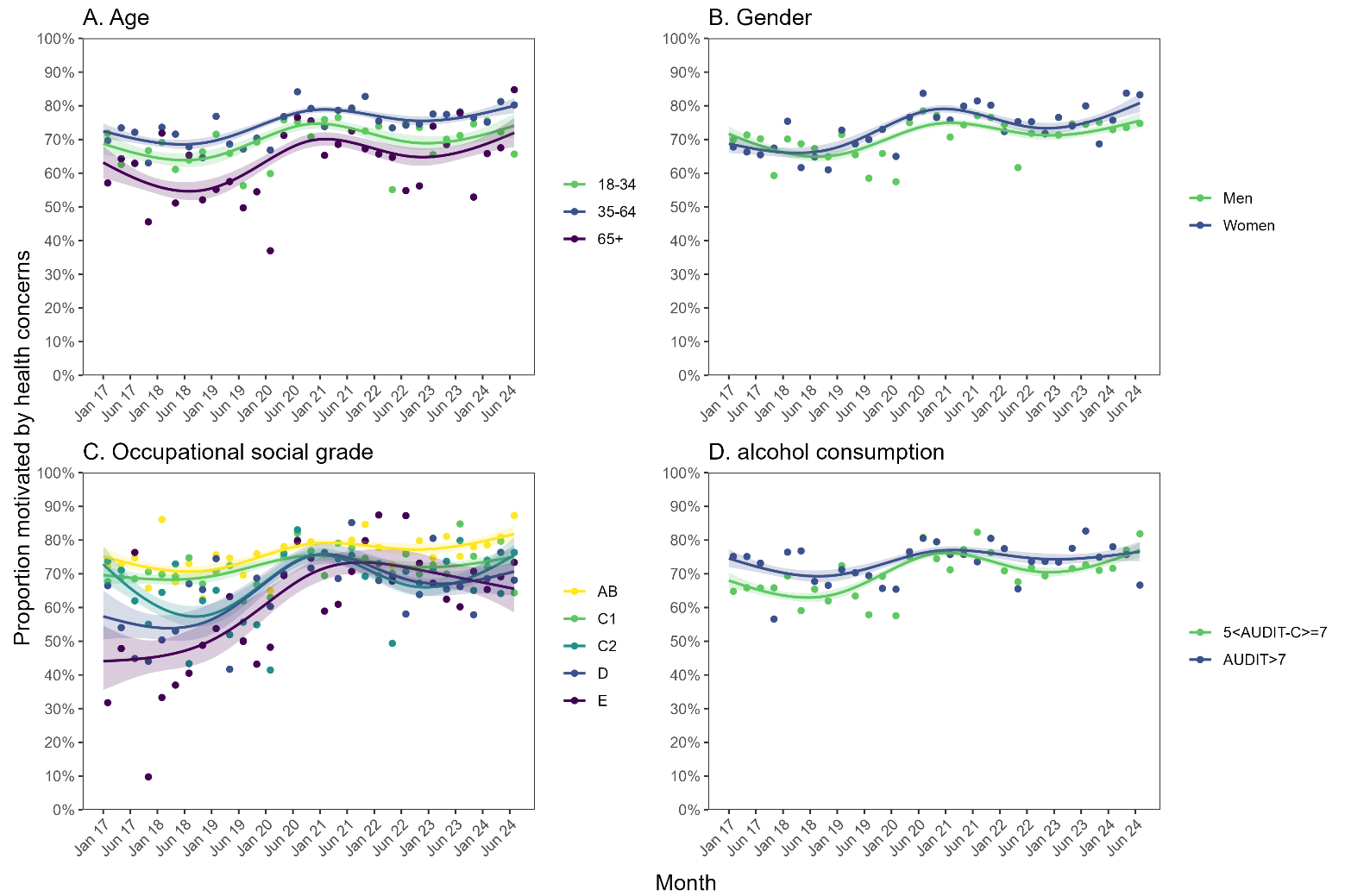


S2 Figure. Time trends in the proportion of attempt to reduce alcohol consumption by health concerns by (A) age (interaction p=0.862), (B) gender (interaction p=0.045), (C) occupational social grade (interaction p=0.028), (D) alcohol consumption (interaction p=0.039), January/2017 to August/2024. Lines represent modelled weighted prevalence by survey month, modelled non-linearly using restricted cubic splines (five knots). Shaded bands represent standard errors. Points represent observed quarterly weighted prevalence.


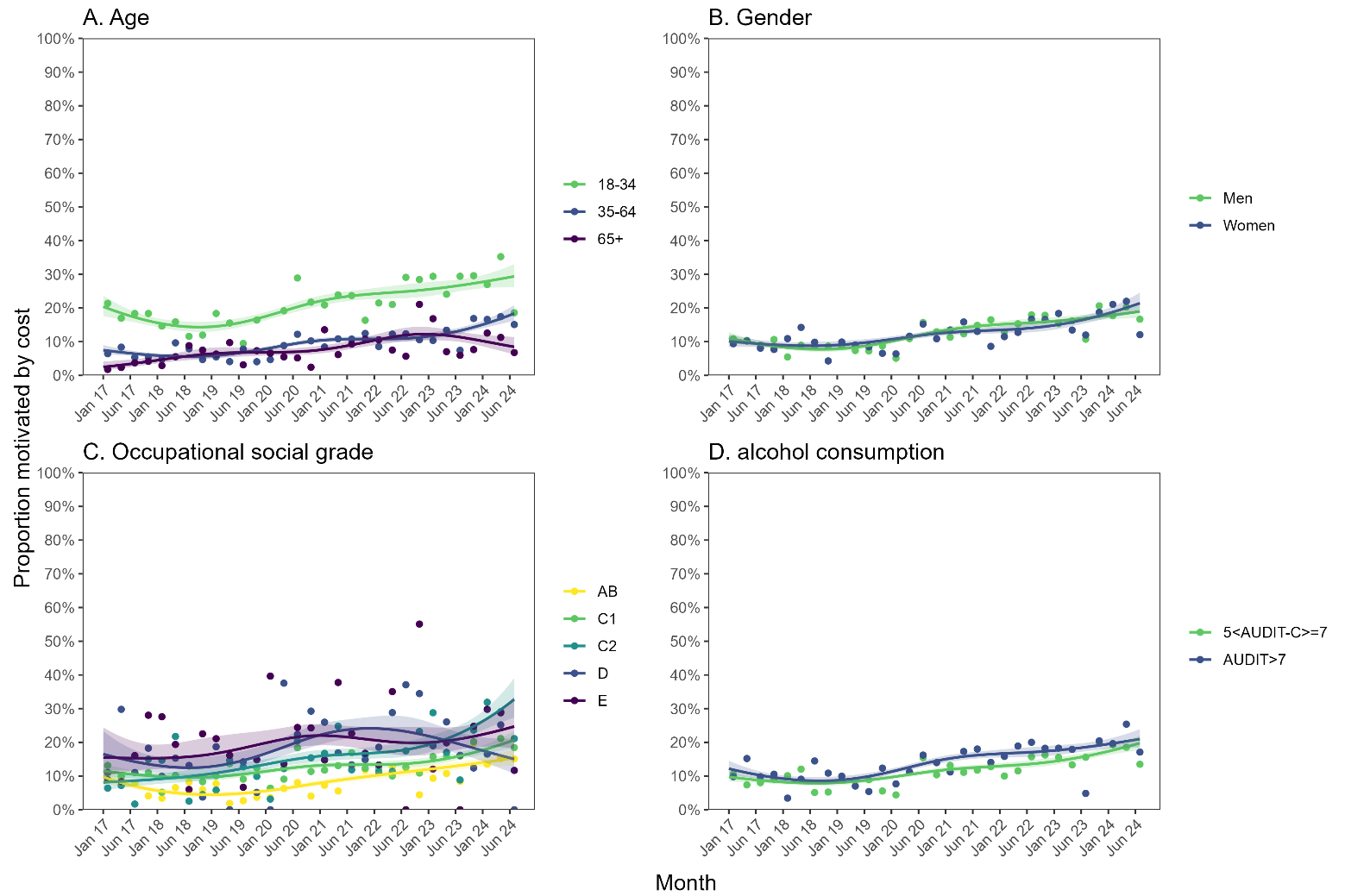


S3 Figure. Time trends in the proportion of attempt to reduce alcohol consumption by cost by (A) age (interaction p=0.041), (B) gender (interaction p=0.705), (C) occupational social grade (interaction p=0.090), (D) alcohol consumption (interaction p=0.860), January/2017 to August/2024. Lines represent modelled weighted prevalence by survey month, modelled non-linearly using restricted cubic splines (five knots). Shaded bands represent standard errors. Points represent observed quarterly weighted prevalence.


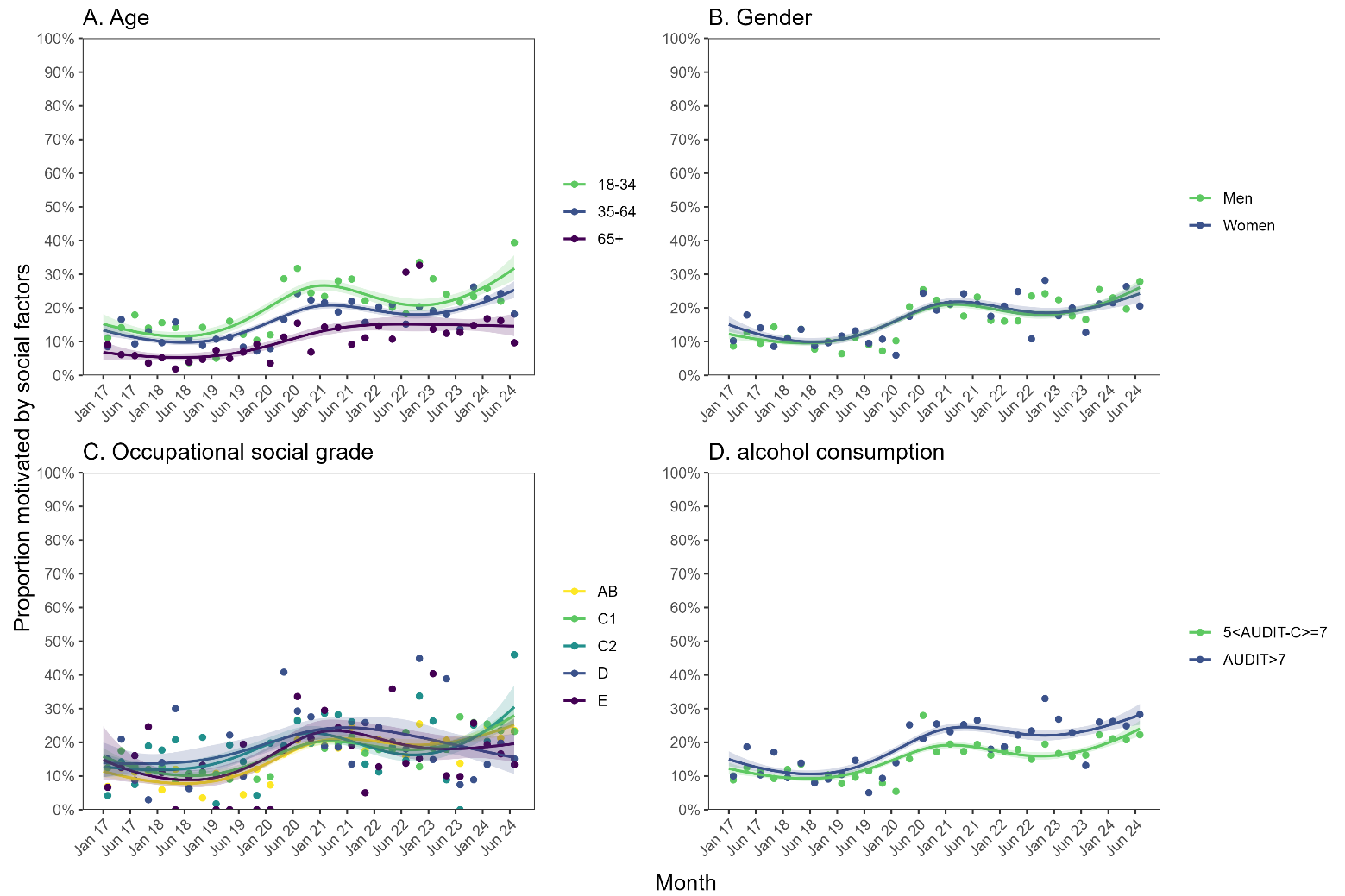


S4 Figure. Time trends in the proportion of attempt to reduce alcohol consumption by social factors by (A) age (interaction p=0.650), (B) gender (interaction p=0.860), (C) occupational social grade (interaction p=0.340), (D) alcohol consumption (interaction p=0.750), January/2017 to August/2024. Lines represent modelled weighted prevalence by survey month, modelled non-linearly using restricted cubic splines (five knots). Shaded bands represent standard errors. Points represent observed quarterly weighted prevalence.


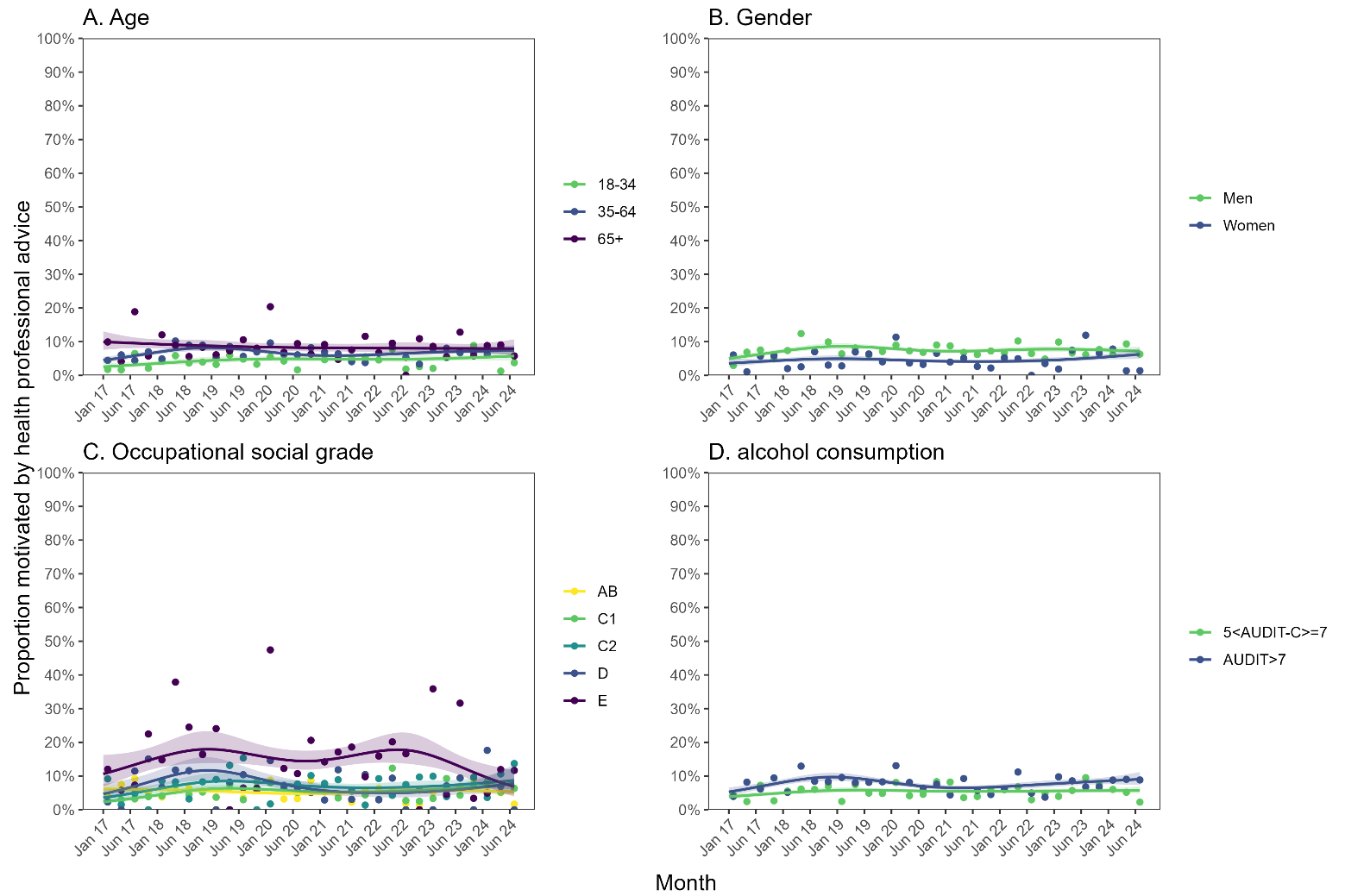


S5 Figure. Time trends in the proportion of attempt to reduce alcohol consumption by health professional advice by (A) age (interaction p=0.601), (B) gender (interaction p=0.780), (C) occupational social grade (interaction p=0.460), (D) alcohol consumption (interaction p=0.670), January/2017 to August/2024. Lines represent modelled weighted prevalence by survey month, modelled non-linearly using restricted cubic splines (five knots). Shaded bands represent standard errors. Points represent observed quarterly weighted prevalence.
